# The Significance of *Myriophyllum elatinoides* for Swine Wastewater Treatment: Abundance and Community Structure of Ammonia-Oxidizing Microorganisms in Sediments

**DOI:** 10.1371/journal.pone.0139778

**Published:** 2015-10-07

**Authors:** Xi Li, Miaomiao Zhang, Feng Liu, Yong Li, Yang He, Shunan Zhang, Jinshui Wu

**Affiliations:** 1 Key Laboratory of Agro-ecological Processes in Subtropical Regions, Institute of Subtropical Agriculture, Chinese Academy of Sciences, Hunan, 410125, P. R. China; 2 Changsha Research Station for Agricultural & Environmental Monitoring, Institute of Subtropical Agriculture, Chinese Academy of Sciences, Hunan, 410125, P. R. China; 3 Graduate University of Chinese Academy of Sciences, Beijing, 100039, P. R. China; University of Malaya, MALAYSIA

## Abstract

*Myriophyllum elatinoides* was reported to effectively treat wastewater by removing nitrogen (N) and phosphorus (P). However, little is known about the abundance and community structure of ammonia-oxidizing microorganisms associated with *M*. *elatinoides* purification systems. The objective of this research was to characterize the abundance and community structure of ammonia-oxidizing microorganisms in swine wastewater and determine the main nitrogen removal pathways. In this study, five different waters were treated by *M*. *elatinoides* in microcosms for one month. The five waters included tap water (Control), swine wastewater (SW), 50% diluted swine wastewater (50% SW), and two synthetic wastewaters: 200 mg NH_4_^+^-N L^−1^ (200 NH_4_^+^-N) and 400 mg NH_4_^+^-N L^−1^ (400 NH_4_^+^-N). The most dramatic changes were in NH_4_^+^-N and total N (TN) concentrations, with average removal rates of 84% and 90%, respectively, in the treatments containing swine wastewater. On days 7, 14, and 28, the dissolved oxygen (DO) increased by 81.8%, 210.4% and 136.5%, respectively, compared with on day 0, in the swine wastewater. The results also showed that the bacterial *amoA* (AOB) copy numbers in the sediments of the treatments were significantly higher than those of archaeal *amoA* (AOA) copy numbers (*p* = 0.015). In addition, the high DO concentrations in swine wastewater responded well to the high abundance of AOB. The AOA and AOB community distributions were positively related with NO_3_^-^N and were negatively related with DO in swine wastewater treatments. In summary, our experimental results suggested that the *M*. *elatinoides* purification system could improve the activity of ammonia-oxidizing microorganisms and consequently might contribute to the significant N removal from the swine wastewater.

## Introduction

Swine wastewater contains high concentrations of nitrogen (N) and phosphorus (P) and was characterized by high chemical oxygen demand (COD) [1, 2]. Swine wastewater is an increasingly considerable source of excess N and causes contamination of surface and ground water. To reduce the environmental impacts, it is necessary to perform treatment prior to spreading and composting applications. One possible solution for swine wastewater treatment was constructed wetlands (CWs). Previous studies on CWs have shown promise for removing N, P, COD, and heavy metals [3–5]. However, the performance of CWs in wastewater treatment depends on the types of wetlands, plants, matrix, and microbial processes [6]. Flowering reed (*Canna indica*), reed (*Phragmites australis*), Cattail (*Typha angustifolia*), and bulrush (*Schoenoplectus lacustris*) are the most common plants, which were planted on CWs around the world [7].

Most previous studies of CWs have speculated that the N removal mechanisms include sedimentation, volatilization, plant uptake, and among others [8]. Removal of most contaminants is attributed chiefly to microbial activity in CWs [9]. While plant uptake is a minor nitrogen removal pathway, microbial nitrification and denitrification play a vital role in CWs [10]. To a large extent, N is removed by microbial activities associated with nitrification, followed by anoxic denitrification in CWs treated with swine wastewater [11]. Oxidation of ammonium (NH_4_^+^) is of importance step in the global nitrogen-cycling, leading to the formation of nitrate (NO_3_^-^) [12, 13]. The ammonia monooxygenase (AMO) enzyme, catalyzes the conversion of ammonium to hydroxylamine, is encoded by the subunits of *amoA* genes. AMO is in charge of the first step of the process of nitrification [14]. Prosser (1989) [15] reported that ammonia-oxidizing bacteria (AOB) have been found to possess nitrification capacity among prokaryotes. AOB plays a role in natural nitrogen cycling and contributes to the transformation of NH_4_^+^ to nitrite (NO_2_^−^) and last to NO_3_^−^ in nitrification [11]. Recently, archaea were also demonstrated to play a vital role in nitrification. The abundances of AOA were more than AOB in terrestrial, aquatic environments [16–19]. Ammonia-oxidizing Crenarchaeota (AOA) have been cultivated at high temperatures that otherwise inhibit AOB growth [20]. Some research also reported that a few strains of AOA were found, but AOB abundances were not found in several aquatic environments [21–23]. The two ammonia-oxidizing groups can have different contributions to nitrification due to physiological differences [24]. At present, more and more researchers studied *Myriophyllum* treating different type wastewater. *Myriophyllum elatinoides* is a perennial submerged macrophyte or floating plant, and grow in all four seasons. *Myriophyllum* spp. also is widely considered as indicator plants for detecting herbicidal activity [25, 26]. A few investigations reported that *M*. *elatinoides* can efficiently remove N from wastewaters [27, 28]. However, despite AOA and AOB abundance present in the environment, the correlations between AOA and AOB community shifts and nitrification rates in CWs with *M*. *elatinoides* used for swine wastewater treatment is not well understood.

Further research on abundance and community structure of AOA and AOB in sediments is very important for understanding the function of the N cycle in wetlands [29]. In this study of an *M*. *elatinoides* purification system treated with swine wastewater, we analyzed the changes in water chemistry and examined the AOA and AOB community shifts using terminal restriction fragment length polymorphism (T-RFLP). Moreover, the influence of environmental variables, such as NH_4_^+^-N, NO_3_^-^N, TN, and DO, on AOA and AOB communities was investigated.

## Materials and Methods

### Experimental description and operation

The experimental microcosms were conducted in a greenhouse (temperature, 23.5–24.5°C, average temperature, 24°C, moisture, 31.5–33.5%, evaporation rate, 0.1%) at the Institute of Subtropical Agriculture, Chinese Academy of Sciences, Hunan, P. R. China from August 14 to September 11, 2013. Plants grew in natural light. Plastic tanks (length 50 cm; width 40 cm; depth 50 cm) were filled with 10 kg of air-dried paddy soil and one hundred plant shoots (20 cm length) were planted into the soil layer of each tank. Paddy soil was classified as Ultisols in the Soil Taxonomy System of the USA. Plants were incubated with tap water for five days. After the added tap water was completely discharged, 15 L of wastewater was added to each *M*. *elatinoides* microcosm. The experimental treatments included a control (tap water), swine wastewater (SW, NH_4_^+^-N concentration of approximately 400 mg L^−1^), swine wastewater diluted with tap water at a 1:1 (v/v) ratio (50%SW, NH_4_^+^-N concentration of approximately 200 mg L^−1^), 200 mg L^−1^ NH_4_^+^-N (200 NH_4_^+^-N), and 400 mg L^−1^ NH_4_^+^-N solution (400 NH_4_^+^-N). Both the 200 NH_4_^+^-N and the 400 NH_4_^+^-N were prepared with ammonium sulfate. The initial pH of the swine wastewater ranged from 7.85 to 7.94, and the initial pH of the synthetic wastewater ranged from 6.28 to 6.48. Each treatment had three duplicates.

### Sampling from constructed wetlands

At 0, 7, 14, and 28 days, 100 mL water samples were collected from each tank for physicochemical analysis. Meanwhile, five sediment subsamples taken at 0–5 cm in depth were collected by using a stainless steel auger (2 cm diameter), then homogenized to make a combined soil sample for molecular analysis. All sediment samples were immediately layed up ice, transported to the laboratory and stored at -20°C for extracting DNA and analysis of sediment molecular properties.

### Wastewater samples characteristics

Concentrations of NH_4_^+^, NO_2_^−^, and NO_3_^−^ were measured according to the US EPA procedures [30] using a fully automated flow-injection system (FIA-star 5000 analyzer, Foss Tecator, Höganäs, Sweden) in water samples.

### Sediment DNA extraction and quantitative PCR analysis of abundance of AOB, AOA

DNA was extracted from 0.5 g of fresh sediment using a MoBio UltraClean® Soil DNA Isolation Kit (San Diego, CA) [19]. The extracted DNA was diluted 10:1 and stored at -20°C. Real-time PCR analysis of *amoA* was performed according to Shen (2008) [31].

### T-RFLP analysis

The primer pairs for AOB [amoA 1F (5′-GGGGTTTCTACTGGTGGT–3′) and amoA 2R (5′-CCCCTCKGSAAAGCCTTCTTC–3′)] [32] and AOA [Arch-amoAF (5′-STAATGGTCTGGCTTAGACG–3′) and Arch-amoAR (5′-GCGGCCATCCATCTGTATGT–3′)] [33] were used for PCR amplification of AOB and AOA genes, respectively. The forward primers were labeled with 6-carboxyfluorescein at the 5′ ends. Reactions were performed in a volume of 50 μL with 25 μL PCR mix (Tiangen, China), 0.2 μmol L^−1^ forward and reverse primer, and approximately 50 ng of template DNA. The amplification reaction for AOB and AOA genes was carried out as follows: 3 min at 95°C, 10 cycles of 95°C for 30 s, 30 s at 65–55°C with a 1°C decrease per cycle, 45 s at 72°C, 30 cycles of 95°C for 45 s, 30 s at 55°C, 40 s at 72°C, and a final extension at 72°C for 10 min. The PCR products were confirmed by a 1.0% agarose gels electrophoresis. Replicate amplicons were purified using a Wizard SV Gel and PCR CleanUp System (Promega, USA). Approximately 200 ng of each amplicon was digested with 5 U of restriction enzyme. *Tag I* and *Hha I* were used for AOA fragments and *msp I* and *Hha I* were used for AOB. The digested products were run on a 3730xl Genetic Analyzer (ABI).

### Statistical analysis

RDA, the direct gradient analysis, was applied to investigate the relationships between the environmental parameters of the *M*. *elatinoides* purification system and AOA and AOB community structures. Multivariate analysis of variance followed by LSD and S-N-K tests were applied to test the differences between treatments, and Pearson’s correlation was applied to test the relationship among days, dissolved oxygen, total nitrogen, NH_4_^+^-N, NO_3_^-^N, and abundance of AOA and AOB. Statistical analysis was carried out using SPSS software version 18.0 (SPSS Inc., Chicago, IL, USA).

## Results

### Wastewater constituents

**Fig 1:**
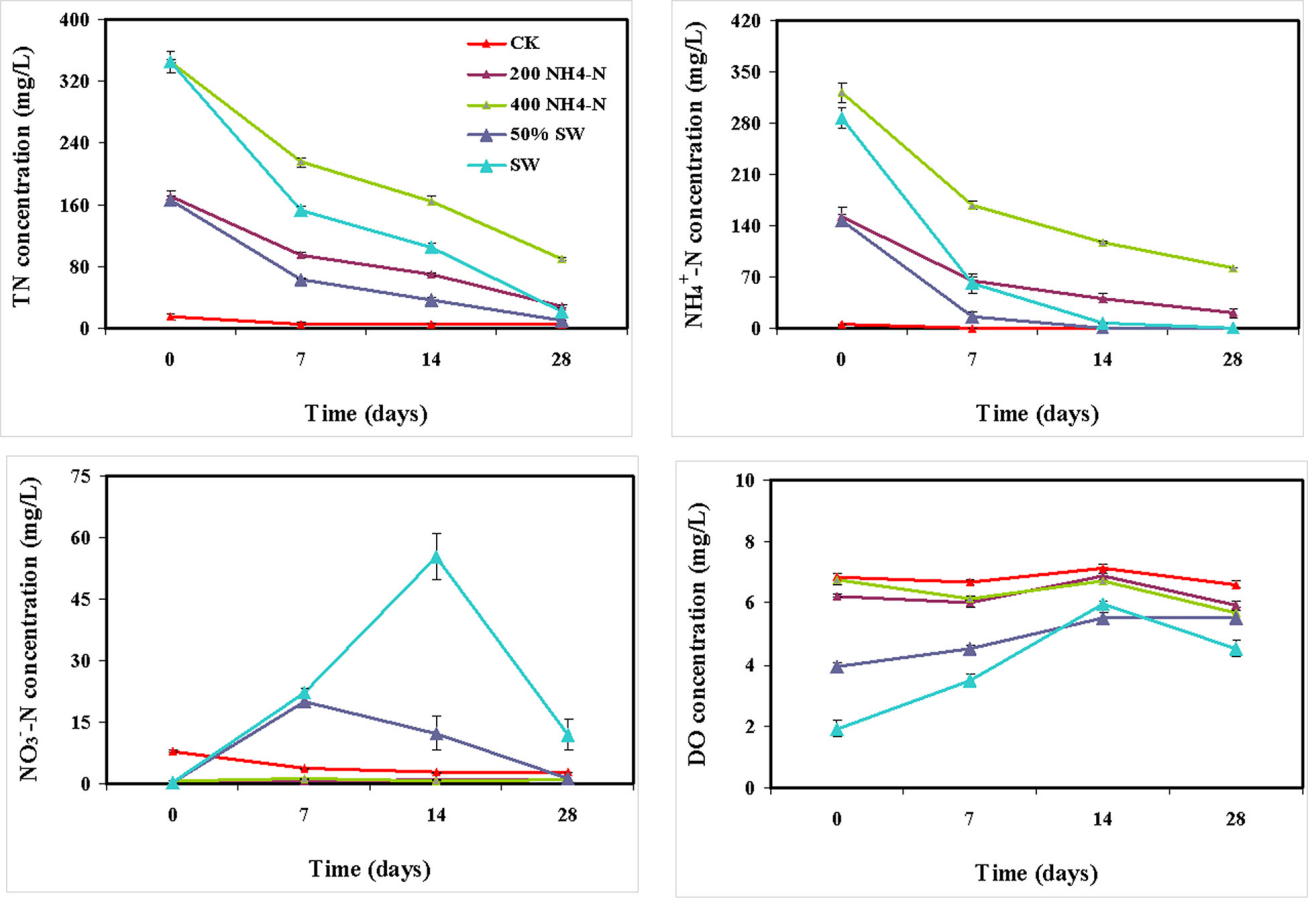
The chemical wastewater characteristics. Small bars show standard errors

Results from the chemical analysis of the wastewater are shown in Fig 1. TN and NH_4_^+^-N concentrations decreased quickly from day 0 to day 7 and more slowly from day 7 to day 28 in all treatments, except for the control. Our results indicate that *M*. *elatinoides* achieved a high N removal efficiency (>90%) over a wide TN concentration range (166–346 mg/L) and a great N removal efficiency (>84%) over a wide NH_4_^+^-N concentration range (147–321 mg/L). NO_3_^-^N concentrations increased quickly from day 0 to day 7 and decreased qucikly from day 14 to day 28 in swine wastewater treatments. The removal efficiencies of the swine wastewater treatments were greater than the synthetic wastewater treatments. DO in swine wastewater increased by 81.8% on day 7, 210.4% on Day 14, and were decreased to 136.5% with sampling time on day 28, respectively, compare with on Day 0. DO in 50% swine wastewater increased by 14.2% on day 7, 40.1% on Day 14, and were decreased to 39.8% with sampling time on day 28, respectively, compare with on Day 0. *M*. *elatinoides* increased the DO concentration in the swine wastewaters.

### Abundance of AOA and AOB

**Fig 2:**
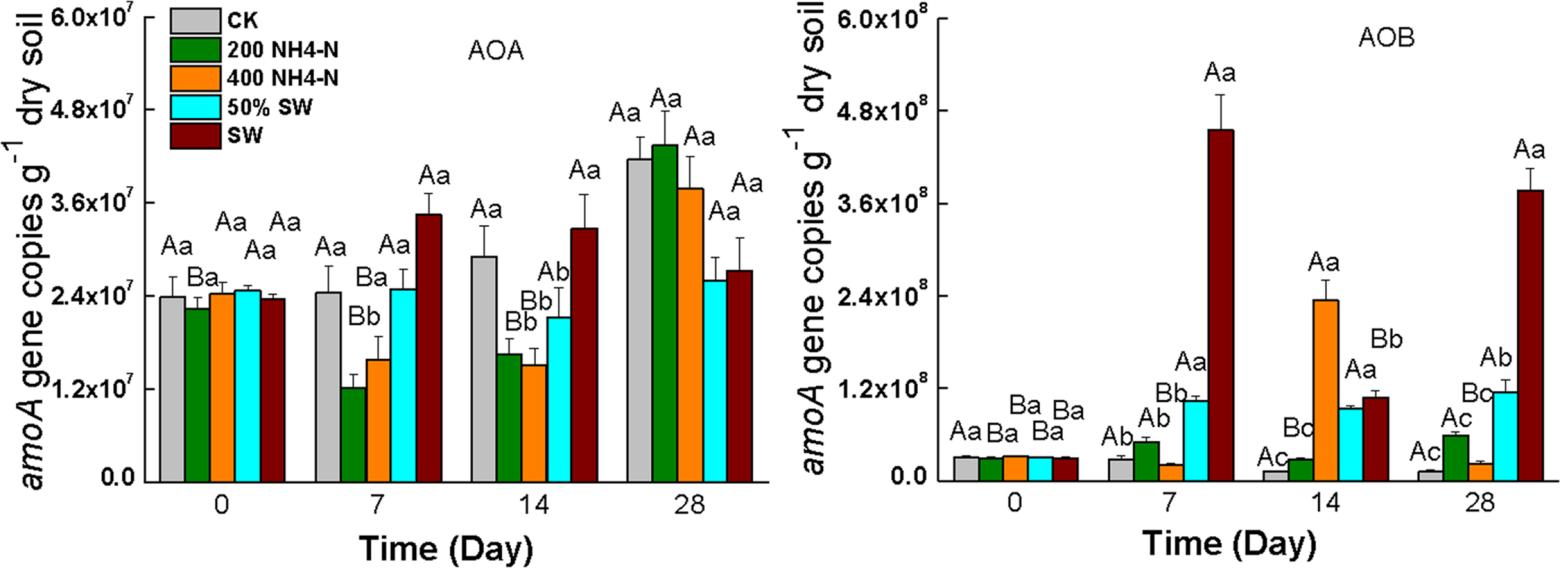
Abundance of AOB and AOA under different days and concentrations treatments. Capital letters indicate the significant differences between differences days in the same concentration treatment (*P*<0.05). Lowercase letters indicate the significant differences between different concentrations treatments in the same day (*P*<0.05). Small bars show standard errors

AOA gene copy numbers were stable in SW treatments (Fig 2). There were no significant differences in abundance of AOA between swine wastewater treatments. In contrast, in the synthetic wastewater treatments (Fig 2), AOA gene copy number decreased on days 7 and 14, and increased on day 28. The archaeal *amoA* genes were 1.21 × 10^7^ to 4.35 × 10^7^ g^−1^ dry soil in different treatments. The bacterial *amoA* copy numbers, ranging from 1.13 × 10^7^ to 4.56 × 10^8^ g^−1^ dry soil, were much higher than the archaeal *amoA* abundance in the different treatments. On day 7 and day 28, the bacterial amoA copy numbers in swine wastewater treatment were significantly higher than those in other treatments, respectively. On day 7, the swine wastewater treatment contained the highest bacterial *amoA* copy numbers, while the 400 NH_4_^+^-N treatment had the lowest.

### T-RFLP profiling

**Fig 3:**
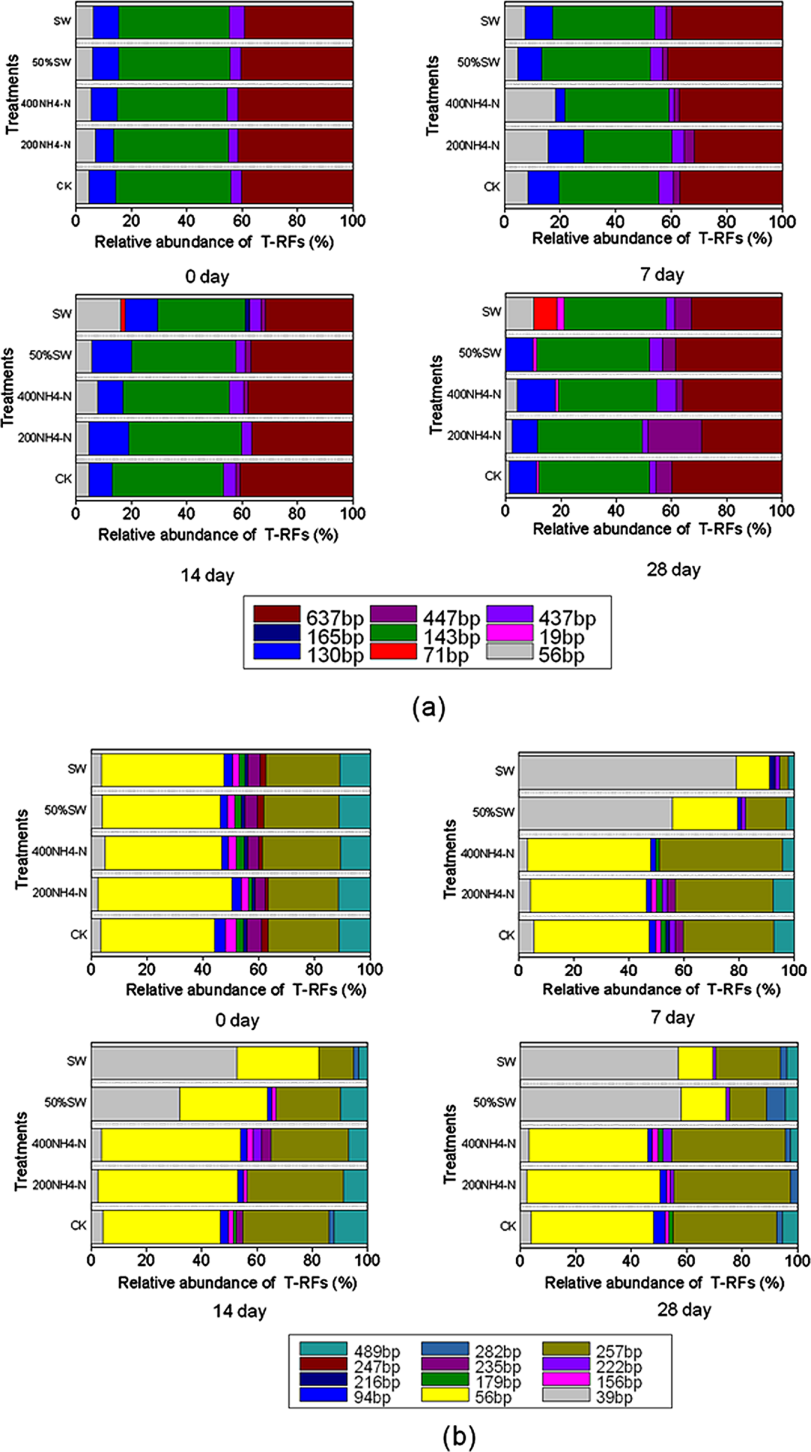
Average relative abundances of AOA (a) T-RFs with endonuclease TaqI and AOB (b) with MspI from different concentrations treatments in different days. The relative abundance of T-RFs is given as a percentage of the total peak height. Fragment sizes within the graph indicate the sizes (bp) of the experimental T-RFs by T-RFLP

A total of 9 different terminal restriction fragments (T-RFs) of the archaeal *amoA* were identified in the different wastewater treatments. T-RFs 71 and 165 were unique to the swine wastewater treatments on days 14 and 28, respectively (Fig 3A). All treatments were composed of the major T-RFs (i.e., 56 bp, 130 bp, 143 bp, 437 bp, 447 bp, and 637 bp). The most abundant and dominant T-RFs found in this study each represented more than 90% of the total peak fluorescence. When comparing all treatments, the swine wastewater treatment differed significantly from the control, 50% swine wastewater, 200 NH_4_^+^-N, and 400 NH_4_^+^-N treatments on days 7 and 14. A total of 12 different T-RFs of the bacterial *amoA* were identified in the different N concentration wastewaters. All treatments were composed of the T-RFs 39, 56, 94, 156, 179, 216, 235, 247, 257 and 489 at day zero (Fig 3B). The 50% and 100% swine wastewater treatments were only composed of the T-RFs 39, 56, 222, 257 and 489 on day 28. The 200 NH_4_^+^-N and 400 NH_4_^+^-N treatments contained, in addition, the T-RF 94 and 156, which could be detected in all synthetic N wastewaters. T-RF 39 was detected at a high percentage (56%–57%) in the swine wastewater treatments, whereas the control and synthetic N wastewater treatments were dominated by T-RF 56 and 257.

**Fig 4:**
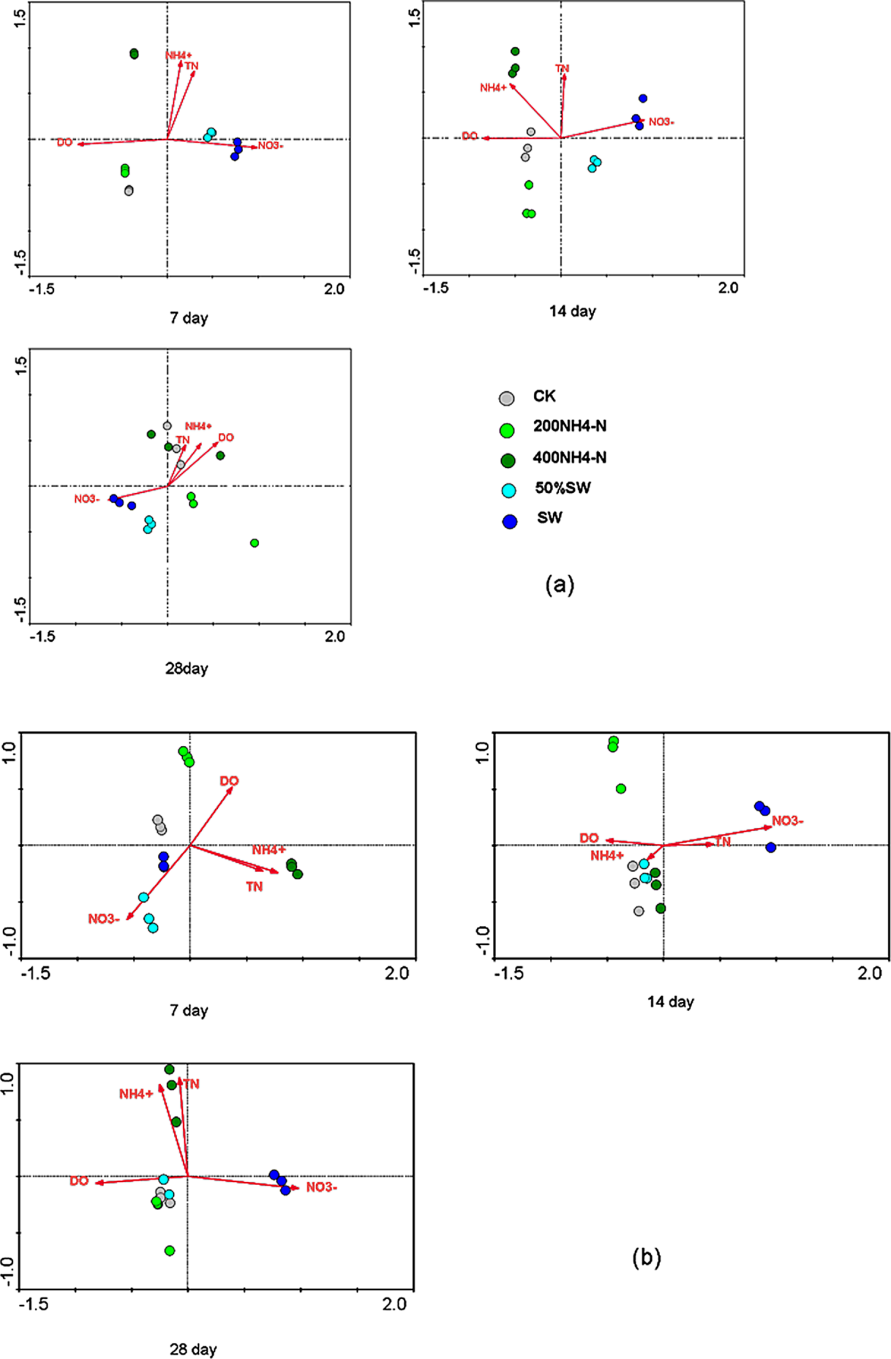
RDA ordination plot of the investigated soil samples, showed water parameters and orientation of samples. The AOA (a) and AOB (b) distributions were based on the relative abundance of T-RFs after restriction using TaqI and MspI enzymes, respectively

RDA was employed to analyze the DO, TN, NH_4_^+^-N and NO_3_^-^N effects on AOA and AOB gene communities (Fig 4). The AOA communities in the swine wastewater treatments and synthetic wastewater treatments were clearly separated from each other at all time points (Fig 4A). The T-RF patterns of the 50% and 100% swine wastewater treatments were positively correlated to NO_3_^-^N, whereas the 200 and 400 NH_4_^+^-N treatments showed a negative correlation with NO_3_^-^N. DO influence the AOA gene compositions in the 200 and 400 NH_4_^+^-N treatments. The AOB community compositions clearly clustered into only two groups in the swine wastewater treatments and synthetic wastewater treatments on day 7 (Fig 4B). Meanwhile, the AOB community compositions in the swine wastewater treatments clustered separately on days 14 and 28. Similar to AOA, combining all environmental parameters in the RDA, AOB in the swine wastewater treatments showed a positive correlation with NO_3_^-^N. DO influence the AOB gene compositions in the 200 and 400 NH_4_^+^-N treatments.

### Pearson’s correlations between nitrification genes and environmental factors

The Pearson’s correlation analysis verified that the AOB gene copy numbers were significantly correlated with DO at different incubation times in the 200 NH_4_^+^-N treatment, 400 NH_4_^+^-N treatment, and the 50% swine wastewater treatment (r = 0.581–0.802, see Table 1). Strong negative correlations were observed between TN and NH_4_^+^-N and AOB gene copy numbers in the 50% swine wastewater treatment and between NO_3_^-^N and AOA gene copy numbers in the swine wastewater treatments.

**Table 1:** Pearson’s correlations between AOA and AOB genes and DO, TN, NH_4_-N and NO_3_-N on Days 0, 7, 14 and 28.

200 NH_4_-N	Days	DO	TN	NH_4_-N	NO_3_-N	AOA	AOB	400 NH_4_-N	Days	DO	TN	NH_4_-N	NO_3_-N	AOA	AOB
Days	1	0.226	-0.959**	-0.885**	-0.911**	0.720**	0.572	Days	1	-0.686*	-0.960**	-0.912**	0.355	0.567	0.066
DO	-0.226	1	0.117	0.009	0.062	-0.517	0.802**	DO	-0.686*	1	0.706*	0.648*	-0.708*	-0.602*	0.581*
TN	-0.959**	0.117	1	0.980**	-0.982**	-0.504	-0.569	TN	-0.960**	0.706*	1	0.989**	-0.567	-0.358	-0.129
NH_4_-N	-0.885**	0.009	0.980**	1	-0.988**	-0.329	-0.519	NH_4_-N	-0.912**	0.648*	0.989**	1	-0.617*	-0.217	-0.219
NO_3_-N	0.911**	0.062	-0.982**	-0.988**	1	0.383	0.433	NO_3_-N	0.355	-0.708*	-0.567	-0.617*	1	-0.111	-0.238
AOA	0.720**	-0.517	-0.504	-0.329	0.383	1	0.468	AOA	0.567	-0.602*	-0.358	-0.217	0.111	1	-0.558
AOB	0.572	-0.802**	-0.569	-0.519	0.433	0.468	1	AOB	0.066	0.581*	-0.129	-0.219	-0.238	-0.558	1
**50%SW**								**SW**							
Days	1	0.900**	-0.922**	-0.847**	-0.197	0.081	0.759**	Days	1	0.675**	-0.967**	-0.867**	0.190	0.108	0.472
DO	0.900**	1	-0.930**	-0.950**	0.003	-0.244	0.751**	DO	0.675*	1	-0.801**	-0.936**	0.851**	0.534	0.194
TN	-0.922**	-0.930**	1	0.975**	-0.189	0.121	-0.927**	TN	0.967**	-0.801**	1	0.957**	-0.381	-0.328	-0.556
NH_4_-N	-0.847**	-0.950**	0.975**	1	-0.281	-0.293	-0.897**	NH_4_-N	-0.867**	-0.936**	0.957**	1	-0.631*	-0.479	-0.455
NO_3_-N	-0.197	0.003	-0.189	-0.281	1	-0.376	0.461	NO_3_-N	0.190	0.851**	-0.381	-0.631*	1	-0.615*	-0.082
AOA	0.081	-0.244	0.121	0.293	-0.376	1	-0.111	AOA	0.108	0.534	-0.328	-0.479	0.615*	1	0.528
AOB	0.759**	0.751**	-0.927**	-0.897**	0.401	-0.111	1	AOB	0.472	0.194	-0.556	-0.455	-0.082	0.528	1

**Significant at *P* < 0.01.

*Significant at *P* < 0.05.

## Discussion

### Nitrogen removal efficiency of *Myriophyllum elatinoides* in wastewater

Our results showed that DO increased when *M*. *elatinoides* was treated with swine wastewater. Such a phenomenon was attributed to the massive *M*. *elatinoides* root system offering a remarkably high oxygen transport capacity by exuding oxygen into the wastewater. The increased DO was beneficial to the nitrification process and ammonium conversion [34]. The *M*. *elatinoides* also had a marked effect on nitrogen removal (90% removal). Previous investigations using CWs with different plant types have shown similar results [35–37]. NO_3_^-^N concentrations increased in beginning experiment stage, the reason could be that ammonium was transformed to large amounts of nitrate in nitration process. DO in swine wastewater increased by an average of 88%. Therefore, *M*. *elatinoides* successfully increased DO concentration and subsequently improved N removal efficiency in the treated wastewater.

### Abundance of AOA and AOB in wastewaters

*amoA* is the indicator gene for aerobic ammonia oxidation and contributes to the oxidation of NH_4_^+^ to NO_3_^-^ during nitrification [38]. There were no significant differences in AOA abundance between the different concentration swine wastewater treatments in the present research, suggesting that the AOA abundance was not a controlling factor for AOA community in the sediment in *M*. *elatinoides* purification system. There were significantly higher AOB copy numbers in the swine wastewater treatments on days 7 and 28 compared with the control and synthetic wastewater treatments (*P* < 0.05). Our results showed that abundances of AOB were more than AOA in the wastewater treatments at all sampling times, suggesting that AOB may play a more vital role than AOA in the nitrification process in wastewater sediments. Our results were inconsistent with other investigations, in which abundances of AOA were more than AOB in marine environments [18, 23, 39, 40], sediments [21, 41], and soils [19, 31]. One of the possible reasons for the high abundance of AOB in our wastewater sediments is the potential for the AOB community to thrive on the episodic NH_4_^+^ fluxes. In ammonium-rich environments, such as the wastewater used in this study, AOB can thrive while AOA may be at a competitive disadvantage. A similar result was reported in a recent study on AOA and AOB in soils [42]. We speculate that *M*. *elatinoides* planted in the wastewater may stimulate the AOB community in the sediments.

On day 7, the abundance of AOB increased to its highest value in swine wastewater treatments, but decreased on day 14 and eventually increased again on day 28, indicating that the ammonium oxidation process was enhanced in the beginning and led the elimination of NH_4_^+^-N. DO in the wastewater was increased by the root system of *M*. *elatinoides*, and therefore the improved oxic conditions in the system favored the growth of AOB [43]. Both AOA and AOB require aerobic conditions for the oxidation of ammonium [44]. In addition, previous studies reported that DO is a vital factor for AOB growth in bioreactors [45]. AOB are enriched by both high DO and low DO in aquatic environments [46]. This might explain the higher abundance of AOB than AOA in the wastewater sediments.

### Impacts of environmental factors on AOA and AOB distribution

The T-RFLP results showed the differences in the AOA and AOB community composition in different treatments. The swine wastewater treatment differed from other treatments in appearance and abundance of the T-RF peaks of archaeal *amoA* on day 28. The swine wastewater treatments differed from the other treatments in appearance and abundance of the T-RF peaks of bacterial *amoA* on days 7, 14 and 28. The T-RFLP analysis also showed a change in community composition in the sediments of wastewater with different N concentrations. Compared with the other treatments, the sediments of the swine wastewater treatment showed a differentiated AOB community composition with the dominance of one specific group, represented by the 39 bp T-RF. In contrast, compared to the control treatment, the sediments of the synthetic wastewater treatments had a similar AOA and AOB community composition, indicating that the synthetic wastewater had little effect on the AOA and AOB community composition. In addition, AOB represented by the 39 bp T-RF might be one of the important contributors to nitrification activity in the swine wastewater treatment. Based on our data, the AOA and AOB community, as determined by the T-RFLP analysis, was primarily affected by the NO_3_^-^N concentrations in the swine wastewater treatments. In the other treatments, the AOA and AOB community was mainly controlled by the DO concentrations in the water. Our results suggested that, in the swine wastewater, AOB nitrifiers were more sensitive to DO than AOA nitrifiers. Meanwhile, we observed that the AOB distribution was influenced by not only the DO concentrations, but also the nitrate concentrations. However, TN and NH_4_^+^-N were not correlated with the AOA and AOB community in the swine wastewater treatments. This might imply that, under the *M*. *elatinoides* purification system, the AOA and AOB gene composition responded to NO_3_^-^N and DO more than to TN and NH_4_^+^-N in the swine wastewater treatments. As time increased, the nitrification process produced more nitrate, and supported the formation of a specialized, uniform community in the sediments of the swine wastewater treatments. These findings demonstrate that the functional communities involved in nitrification responded differently to the environmental changes. The AOB diversity in the swine wastewater treatments was very sensitive to the variable environmental conditions (e.g. DO, ammonium). These results are supported by many studies of AOB population distribution in constructed wetlands [47–49]. The diversity of AOB in the sediments of swine wastewater treatments in this study was higher than that in sewage/industrial wastewaters and in marine water [46, 50]. This was probably due to the *M*. *elatinoides* purification system having effects on the microbial communities in the sediments.

### Impacts of environmental factors on abundance of AOA and AOB

The Pearson’s correlation coefficients between DO and abundance of AOB were greater than 0.58 (*P* < 0.05), confirming an interaction between environmental factors and microbial communities in all treatments except the swine wastewater treatment. Coolen [51] deduced that the abundance of archaeal *amoA* might form a vital source of nitrite for the anammox at very low oxygen conditions in the Black Sea. We found a negative correlation between DO and abundance of AOA, except in the swine wastewater treatment, which may suggest that AOA can become accustomed to growth at low oxygen levels. The ammonium concentration was one of the major factors influencing abundance and distribution of AOA. In agreement with our results, several investigations stated that AOA abundance is negatively correlated with ammonium concentration in wastewater treatment plants [52, 53]. Overall, our data showed that AOA can very likely adapt to an environment with low ammonium concentration and availability.

## Conclusion

Our results showed that the *M*. *elatinoides* purification system can improve dissolved oxygen in the wastewater, and thus can help to stimulate the nitrification process. The *M*. *elatinoides* purification system can achieve high and stable NH_4_^+^-N (>84%) and TN (>90%) removal efficiencies without aeration. The quantitative analyses confirmed the increasing abundance of AOB in the system, which is useful for increasing N removal. Our results further revealed that the AOB-dominated nitrification process was the main N removal pathway in the system. DO and NO_3_^-^N were the key environmental factors influencing AOA and AOB distribution. An increase in DO is predicted to support and sustain sediment nitrification while an increase in NO_3_^-^N may inhibit the nitrification process in sediments. The Pearson’s correlation analysis showed a strong positive correlation between DO and abundance of AOB suggesting that DO was coupled, at the molecular level (abundance of functional genes), to contribute to N removal in the system. Our study demonstrated that the *M*. *elatinoides* purification system not only contributed to direct N removal by plant uptake, but also stimulated the ammonia-oxidizing activity of the microorganisms in the wastewater sediments via transporting oxygen to the wastewater through its root system.
